# Metabolomics for Biomarker Discovery in Gastroenterological Cancer

**DOI:** 10.3390/metabo4030547

**Published:** 2014-07-07

**Authors:** Shin Nishiumi, Makoto Suzuki, Takashi Kobayashi, Atsuki Matsubara, Takeshi Azuma, Masaru Yoshida

**Affiliations:** 1 Division of Gastroenterology, Department of Internal Medicine, Kobe University Graduate School of Medicine, 7-5-1, Kusunoki-cho, Chu-o-ku, Kobe, Hyogo 650-0017, Japan; E-Mails: msuzuki@med.kobe-u.ac.jp (M.S.); kobatak@med.kobe-u.ac.jp (T.K.); amtbr@med.kobe-u.ac.jp (A.M.); azumat@med.kobe-u.ac.jp (T.A.); 2 Division of Metabolomics Research, Department of Internal Related, Kobe University Graduate School of Medicine, 7-5-1, Kusunoki-cho, Chu-o-ku, Kobe, Hyogo 650-0017, Japan

**Keywords:** metabolomics, biomarker, serum, gastroenterological cancer, mass spectrometry

## Abstract

The study of the omics cascade, which involves comprehensive investigations based on genomics, transcriptomics, proteomics, metabolomics, *etc.*, has developed rapidly and now plays an important role in life science research. Among such analyses, metabolome analysis, in which the concentrations of low molecular weight metabolites are comprehensively analyzed, has rapidly developed along with improvements in analytical technology, and hence, has been applied to a variety of research fields including the clinical, cell biology, and plant/food science fields. The metabolome represents the endpoint of the omics cascade and is also the closest point in the cascade to the phenotype. Moreover, it is affected by variations in not only the expression but also the enzymatic activity of several proteins. Therefore, metabolome analysis can be a useful approach for finding effective diagnostic markers and examining unknown pathological conditions. The number of studies involving metabolome analysis has recently been increasing year-on-year. Here, we describe the findings of studies that used metabolome analysis to attempt to discover biomarker candidates for gastroenterological cancer and discuss metabolome analysis-based disease diagnosis.

## 1. Introduction to Metabolomics

### 1.1. Omics

The study of the omics cascade, which involves comprehensive investigations based on genomics, transcriptomics, proteomics, metabolomics, *etc.*, has developed rapidly and now plays an important role in life science research. Genomics makes it possible to find gene mutations and gene polymorphisms, and there is an abundance of reports about genomics-based studies. Transcriptomics is an approach in which biological samples are analyzed to obtain information about the concentrations of a large number of mRNA transcripts in a simultaneous manner. The latter information includes gene expression data related to functional genomics. Recently, the comprehensive analysis of microRNA molecules has attracted increasing attention in the life science field, especially the medical research field. In proteomics, information about protein expression levels and functions, such as about abnormal protein expression, protein phosphorylation, and protein interactions, is evaluated, and a great number of academic papers about such research have been published. Recently, metabolomics or metabolome analysis, which involves the comprehensive analysis of low molecular weight metabolites, has rapidly developed along with improvements in analytical technology leading to its use in a variety of research fields including clinical, cell biology, and plant/food science studies [1,2,3,4].

### 1.2. Characteristics of Metabolomics

Metabolome analysis is used to evaluate the characteristics and interactions of low molecular weight metabolites under a specific set of conditions, for example, at a particular developmental stage, in certain environmental conditions, or after specific genetic modifications. The metabolome mainly represents the endpoint of the omics cascade and is also the closest point in the cascade to the phenotype. Changes in metabolite levels can also be induced by exogenous factors, such as environmental and dietary factors, while genomic information is not basically affected by such factors. Moreover, in addition to expression variations, the metabolome is also affected by the enzymatic activities of various proteins. Therefore, metabolite profiles are considered to be a summary of the other upstream omics profiles, and metabolome analysis might be able to detect subtle changes in metabolic pathways and deviations from homeostasis before phenotypic changes occur [5,6]. Taken together, the metabolite profile of a cell is more likely to represent the cell’s status than its DNA, RNA, or protein profile. On the basis of these facts, metabolomics has started to be used in medical research. In such studies, metabolomics has mainly been utilized to discover biomarker candidates for cancer. For example, a search of the papers included in the PubMed database [7] using the keywords “metabolomics”, “cancer”, and “biomarker” found a total of 377 papers, and the number has been increasing year-on-year. In addition, metabolomics can also be used to examine unknown pathological conditions. Here, we describe the findings of studies that have used metabolome analysis to attempt to discover biomarker candidates for gastroenterological cancer and discuss metabolome analysis-based disease diagnosis.

## 2. Metabolism in Cancer

D. Hanahan and R.A. Weinberg suggested that the following characteristics are hallmarks of cancer: sustained proliferation, evasion from growth suppressors, active invasion and metastasis, the enabling of replicative immortality, the induction of angiogenesis, resistance to cell death, the deregulation of cellular energetics, avoidance of immune destruction, tumor-promoting inflammation, and genome instability and mutation [8,9]. Among these characteristics, the deregulation of cellular energetics seems to be particularly related to metabolomics research. Actually, in the study by Hirayama *et al.,* metabolome analysis using capillary electrophoresis-mass spectrometry (CE-MS) demonstrated that colon and gastric tumors produce energy by glycolysis rather than oxidative phosphorylation via the tricarboxylic acid cycle, even in the presence of an adequate oxygen supply, which is known as “the Warburg effect” [10]. The accumulation of significantly higher levels of most amino acids, which are indirectly used as energy sources, in tumor tissue compared with non-tumor tissue has also been reported [10]. In addition, cancer cells obtain energy via glutaminolysis as well as glycolysis [11]. The relationship between “active and metastasis” and metabolites has started to be elucidated. Sarcosine, which is an N-methyl derivative of the amino acid glycine, was identified as a potentially important metabolic intermediary for prostate cancer cell invasion and aggressivity [12]. It was also proposed that glycine metabolism may represent a metabolic vulnerability in rapidly proliferating cancer cells [13]. Thus, metabolites themselves seem to affect cancer cells somehow, and these findings suggest that the pathogenesis of cancer leads to alterations in metabolite levels in the body. If such metabolite alterations influence the metabolite levels in biological fluids such as serum/plasma, urine, and saliva, it may be possible to use the metabolite concentrations of such fluids as biomarkers of cancer.

## 3. Biomarker Discovery in Gastroenterological Cancer Using Metabolomics

### 3.1. Biomarker Discovery and Gastroenterological Cancer

Gastroenterological cancer is a group of cancers including esophageal, gastric, colorectal, hepatic, and pancreatic cancer. Oral cancer may be also included in gastroenterological cancer. Gastroenterological cancer remains relatively asymptomatic until it reaches the progressive state, at which point it exhibits poor prognosis. Therefore, methods that facilitate the detection of gastroenterological cancer at an earlier stage are desired, because early stage cancer patients are highly likely to make a complete recovery from such conditions. Regarding gastroenterological cancer, biomarkers that make it possible to accurately predict prognosis, therapeutic efficacy, and adverse effects are also required. Recently, studies on metabolomics-based biomarker discoveries have been widely reported (Table 1). In addition, there are some articles in which the metabolite alterations in tumor tissues were evaluated using tissue metabolome analysis and the results leading to elucidation of pathogenesis of gastroenterological cancer were shown (Table 1). The pathogenesis of gastroenterological cancer is considered to be closely associated with lifestyle factors as well as genome mutations associated with oncogenes and tumor suppressor genes. Therefore, metabolomics is likely to be a suitable method for biomarker discovery [14], as described in the “Introduction to Metabolomics”.

### 3.2. Metabolomics-Based Biomarker Discovery

Metabolomics-based biomarker discovery studies for gastroenterological cancer have been widely performed by liquid chromatography-mass spectrometry (LC-MS), gas chromatography-mass spectrometry (GC-MS), CE-MS, nuclear magnetic resonance (NMR) spectroscopy or Fourier transform-infrared (FT-IR) spectroscopy (Table 1). MS-based techniques exhibit relatively high selectivity and sensitivity during such analyses, and therefore, they have often been used for metabolite profiling. In metabolite profiling, analyses have been performed focusing on the metabolites related to the specific pathways, e.g., amino acids, organic acids, carbohydrates, and lipids [15], and then precise identification of the metabolites and correction for analytical inaccuracies are needed [16]. On the contrary, metabolic fingerprinting is the method for sample classification, and the target samples’ spectral patterns based on their biological state and/or origin are available [17]. NMR and FT-IR spectroscopy display relatively low selectivity so they are often used in metabolite fingerprinting aimed at evaluating the differences among biological samples, although there have been some studies in which metabolite profiling was performed using NMR and FT-IR. Therefore, metabolite profiling and metabolite fingerprinting are applied to metabolomics-based biomarker discovery.

### 3.3. Biomarker Discovery in Gastroenterological Cancer by Metabolomics

Previous studies about metabolome analysis in patients with gastroenterological cancer have analyzed the metabolites present in serum/plasma, saliva, urine, feces, and/or tissue samples, and there is a particular abundance of reports about the metabolites found in serum/plasma samples. Recent reports about metabolome analysis in patients with gastroenterological cancer are listed in Table 1. Metabolome analysis-based attempts to elucidate biomarker candidates for gastroenterological cancer have been carried out using a variety of techniques including GC-MS, LC-MS, CE-MS, NMR, and Fourier transform ion cyclotron resonance (FTICR)-MS. Each approach has different characteristics, and it is impossible to measure all metabolites including hydrophobic and hydrophilic molecules using a single technique. Therefore, in some biomarker discovery studies, a variety of instruments were used, which allowed the researchers to evaluate the potential of a wide range of metabolites as novel biomarkers. However, there were some inconsistencies between the results obtained by different research groups. For example, in the report by Chen *et al.* the urine level of isoleucine was higher in colorectal cancer patients compared with healthy controls [18]. On the contrary, Qiu *et al.* demonstrated that colorectal cancer patients had lower urinary levels of isoleucine [19]. In addition, differences between the presence and absence of significant alterations in metabolite concentrations have also been observed. These discrepancies might have been due to the differences in the methods used to collect the biological samples, and these issues are discussed in Section 4. Thus, although there is an abundance of reports about the use of metabolome analysis to discover biomarker candidates for gastroenterological cancer, no firm conclusions have yet been reached.

**Table 1:** A list of recent reports in which patients with gastroenterological cancer were subjected to metabolome analysis.

Disease	Specimen	Upregulated Metabolites	Downregulated Metabolites	Analytical Method	Ref.
Oral cancer	Saliva	Lactate; *n*-Eicosanoate	Valine; GABA; Phenylalanine	UPLC-Q-TOF/MS	[20]
**Research aim:** To discover salivary metabolite biomarkers and to explore salivary metabolomics as a disease diagnostic tool					
Oral cancer	Saliva	Cadaverine; 2-Aminobutyrate; Alanine; Piperidine; Taurine; Piperideine; Pipecolate; Pyrroline hydroxycarboxylate; Betaine; Leucine + Isoleucine; Phenylalanine; Tyrosine; Histidine; Valine; Tryptophan; β-Alanine; Glutamate; Threonine; Serine; Glutamine; Choline; Carnitine	None	CE-TOF-MS	[21]
**Research aim:** To predict oral cancer susceptibility via saliva-based diagnostics based on metabolomics technology					
Oral cancer	Urine	Alanine; Valine; Serine; Tyrosine; Cystine	6-Hydroxynicotinate; Hippurate	GC-QMS	[22]
**Research aim:** To establish a diagnostic tool for early stage oral squamous cell carcinoma and its differentiation from other oral conditions by the urinary metabolite profiling approach					
Oral cancer	Serum	Glycerate; Serine; Laurate; *N*-Acetyl-l-aspartate; Asparagine; Ornithine; Heptadecanate	None	GC-QMS	[23]
**Research aim:** To find metabolite biomarker candidates for detection of early stage oral squamous cell carcinoma					
Esophageal cancer	Mucosal tissue	l-Valine; Naphthalene; 1-Butanamine; Pyrimidine; Aminoquinoline; l-Tyrosine; Isoleucine; Purine; Serine; Phosphate; myo-Inositol; Arabinofuranoside; l-Asparagine; Tetradecanoate; l-Alanine; Hexadecanoate	l-Altrose; d-Galactofuranoside; Arabinose; Bisethane	GC-QMS	[24]
**Research aim:** To find tissue metabolomic biomarkers that are identifiable and diagnostically useful for esophageal cancer					
Esophageal cancer	Mucosal tissue	*N*-acetylaspartate; Glutamate; Valine; Leucine + Isoleucine; Tyrosine; Methionine; Phenylalanine; GABA; Phenylacetylglutamine; Glutamic acid γ-H; Unsaturated lipids; Short-chain fatty acids; Phosphocholine; Glycoproteins; Acetone; Malonate; Acetoacetate; Acetate; Trimethylamine; Formate; Uracil; Adenine in ATP/ADP and NAD/NADH; Acetyl hydrazine; Hippurate	Creatine; Glycine; Glutamine; 4-Hydroxyphenylpyruvate; Creatinine; Taurine; Aspartate; myo-Inositol; Cholesterol; Choline; Glucose; Ethanol; α-Ketoglutarate oxime; AMP; NAD	NMR	[25]
**Research aim:** To find the potential tissue metabolite biomarkers for clinical diagnosis for different stages of human esophageal cancer and new insights for the mechanism research					
Esophageal cancer	Tissue	Choline; Alanine; Glutamate	Creatinine; myo-Inositol; Taurine	NMR	[26]
**Research aim:** To establish the biochemical profiles of adjacent non-involved tissue and malignant esophageal tumor and to determine the metabolomic changes of tumors with different tumor differentiation for finding metabolomic indicators sensitive to tumor differentiation					
Esophageal cancer	Urine	Urea; Acetate; Pantothenate; 3-Hydroxyisovaleate; Acetone; Formate; 2-Hydroxyisobutyrate; Creatinine; Ethanolamine; 2-Aminobutyrate; Leucine; Succinate; Glutamine; Glucose; Glycine; Tryptophan; Trimethylamine-*N*-oxide; Valine; Lactate; Tyrosine	Dimethylamine; Alanine; Citrate	NMR	[27]
**Research aim:** To find urinary metabolite signatures that can clearly distinguish both Barrett’s esophagus and esophageal cancer from controls					
Esophageal cancer	Serum	Uridine	1-Methyladenosine; *N*^2^,*N*^2^-Dimethylguaosine; *N*^2^-Methylguanosine; Cytidine	LC-QqQ/MS	[28]
**Research aim:** To investigate whether nucleosides can potentially serve as useful biomarkers to identify esophageal adenocarcinoma					
Esophageal cancer	Serum	Lactate; β-Hydroxybutyrate; Lysine; Glutamine; Citrate	Valine; Leucine + Isoleucine; Methionine; Tyrosine; Tryptophan; Myristate; Linoleate	LC-Q-TOF/MS NMR	[29]
**Research aim:** To identify the metabolite based biomarkers associated with the early stages of esophageal adenocarcinoma with the goal of improving prognostication					
Esophageal cancer	Serum	β-Hydroxybutyrate; Acetoacetate; Creatine; Creatinine; Lactate; Glutamate; Glutamine; Histine	LDL/VLDL; Unsaturated lipids; Acetate; α-Glucose; β-Glucose; Tyrosine	NMR	[30]
**Research aim:** To characterize the systemic metabolic disturbances underlying esophageal cancer and to identify possible early biomarkers for clinical prognosis					
Esophageal cancer	Serum	Lactate; Glycolate; Malonate; Fumarate; l-Serine; l-Aspartate; l-Glutamine	Pyruvate	GC-QMS	[31]
**Research aim:** To investigate the differences in serum metabolite profiles using a metabolomic approach and to search for sensitive and specific metabolomic biomarker candidates					
Esophageal cancer	Plasma	Phosphatidylinositol; Lithocholyltaurine; Phosphatidiate; L-Urobilinogen; 9'-Carboxy-γ-tocotrienol; PC; PE; Sphinganine 1-phosphate; Phosphatidylserine(16:0/14:0); LPC(22:2); Ganglioside GM2(d18:1/24:1(15Z)); Lithocholate 3-*O*-glucuronide; 12-Oxo-20-dihydroxy-leukotriene B4	Desmosine; Isodesmosine; 5-β-Cyprinol sulfate	UPLC-TOF/MS	[32]
**Research aim:** To search for valuable markers including circulating endogenous metabolites associated with the risk of esophageal cancer					
Gastric cancer	Tissue	2-Aminobutyrate; 3-Aminoisobutanoate; Valine; 2-Hydroxy-4-methyl-pentanoate; Isoleucine; Proline; Uracil; Threonine; Thymine; Dihydrouracil; Aspartate; Pyroglutamate; GABA; Cysteine; Glutamate; Dodecanoate; Asparagine; Putrescine; Cadaverine; Ascorbate; Gluconate; Xanthine; *N*-Acetyl glucosamine; Kynurenine; Inosine	Hydroxyacetate; 3,4-Dihydroxy-2(3H)-furanone; Nicotinamide; Glycerol phosphate; Tetradecanoate; Palmitelaidate; Palmitate; Linoleate; Stearate; Arachidonate; l-Palmitoyl-glycerol; Sucrose; Cholesterol	GC-TOF/MS	[33]
**Research aim:** To reveal the major metabolic alterations essential for the development of gastric cardia cancer and to discover a biomarker signature of gastric cardia cancer					
Gastric cancer	Urine	Arginine; Leucine; Valine; Isoleucine; Lactate	Methionine; Serine; Aspartate; Histidine; Succinate; Citrate; Malate	CE-MS	[34]
**Research aim:** To search for potential tumor markers of gastric cancer in patients’ urine samples					
Gastric cancer	Serum	3-Hydroxypropionate; 3-Hydroxyisobutyrate	Pyruvate; Octanoate; Phosphate	GC-QMS	[31]
**Research aim:** To investigate the differences in serum metabolite profiles using a metabolomic approach and to search for sensitive and specific metabolomic biomarker candidates					
Gastric cancer	Serum	*L*-Valine; Sarcosine; Hexadecanenitrile	*L*-Glutamine; Hexanedioate; 9,12-Octadecadienoate; 9-Octadecenoate; trans-13-Octadecenoate; Nonahexacontanoate; Cholesta-3,5-diene; Cholesterol/Pentafluoropropionate; Cholesterol; Cholest-5-en-3-ol; Fumarate; 2-*o*-Mesyl arabinose; Benzeneacetonitrile; 2-Amino-4-hydroxy-pteridinone; 1,2,4-Benzenetricarboxylate	GC-QMS	[35]
**Research aim:** To explore the underlying metabolic mechanisms of gastric cancer and to identify biomarkers associated with morbidity					
Colorectal cancer	Mucosal tissue	Lactate; Phosphate; l-Glycine; 2-Hydroxy-3-methylvalerate; l-Proline; L-Phenylalanine; Palmitate; Margarate; Oleate; Stearate; Uridine; 11,14-Eicosadienoate; 11-Eicosenoate; 1-*o*-Heptadecylglycerol; 1-Monooleoylglycerol; Propyl octadecanoate; Cholesterol	Fumarate; Malate; d-Mannose; d-Galactose; d-Glucose; 1-Hexadecanol; Arachidonate	NMR GC-QMS	[36]
**Research aim:** To reveal that global metabolic profiling of colon mucosae can define metabolic signatures for not only discriminating malignant from normal mucosae but also distinguishing the anatomical and clinicopathological characteristics of colorectal cancer					
Colorectal cancer	Tissue	Glycine; l-Proline; l-Phenylalanine; l-Alanine; l-Leucine; l-Valine; l-Serine; l-Threonine; l-Isoleucine; Picolinate; l-Methionine; l-Aspartate; β-Alanine; Aminomalonate; 1-Methylhydantoin; Palmitate; Margarate; Oleate; Stearate; 11-Eicosenoate; Myristate; Pentadecanoate; Linolenate; Lignocerate; Phosphate; l-Arabinose; Lactate; Maleate; Pantothenate; Glycerol; 1-Monooleoylglycerol; Uracil; Uridine; Cholesterol	Arachidonate; d-Mannose; d-Galactose; d-Glucose; Fumarate; Malate; Oxalate; Succinate; Ribitol; Squalene	GC×GC-TOF/MS	[37]
**Research aim:** To investigate whether the metabotype associated with colorectal cancer is distinct from that of normal tissue and whether various biochemical processes are altered by pathogenesis of colorectal cancer					
Colorectal cancer	Urine	Lactate; Arginine; Leucine; Isoleucine; Valine	Histidine; Methionine; Aspartate; Serine; Succinate; Citrate; Malate	CE-IT/MS	[18]
**Research aim:** To investigate the metabolic profile of urine metabolites and to elucidate their clinical significance in patients with colorectal cancer including possibility as the biomarker candidates for early detection.					
Colorectal cancer	Urine	5-Hydroxytryptophan; 5-Hydroxyindoleacetate; Tryptophan; Glutamate; Pyroglutamate; *N*-Acetyl-aspartate; *p*-Cresol; 2-Hydroxyhippurate; Phenylacetate; Phenylacetylglutamine; *p*-Hydroxyphenylacetate	Succinate; Isocitrate; Citrate; 3-Methylhistidine; Histidine	GC-QMS	[19]
**Research aim:** To demonstrate the potentials of this noninvasive urinary metabolomic strategy as a complementary diagnostic tool for colorectal cancer					
Colorectal cancer	Serum	None	FAs (C_28_H_46_O_4_, C_28_H_48_O_4_, C_28_H_50_O_4_)	FTICR-MS LC-Q-TOF/MS NMR QqQ-MS	[38]
**Research aim:** To discover putative metabolomic markers associated with colorectal cancer					
Colorectal cancer	Serum	Pyruvate; α-Hydroxybutyrate; Phosphate; Isoleucine; β-Alanine; meso-Erythritol; Aspartate; Pyroglutamate; Glutamate; *p*-Hydroxybenzoate; Arabinose; Asparagine; Xylitol; Ornithine; Citrulline; Glucuronate; Glucosamine; Palmitoleate; Inositol; Kynurenine; Cystamine; Cystine; Lactitol	Nonanoate; Creatinine; Ribulose; *o*-Phosphoethanolamine	GC-QMS	[39]
**Research aim:** To establish new screening methods for early diagnosis of colorectal cancer via metabolomics					
Colorectal cancer	Serum	Lactate; Glycolate; l-Alanine; 3-Hydroxypropionate; l-Proline; L-Methionine; Thioglycolate; l-Glutamate; l-Asparagine; l-Glutamine; Glucuronic lactone	None	GC-QMS	[31]
**Research aim:** To investigate the differences in serum metabolite profiles using a metabolomic approach and to search for sensitive and specific metabolomic biomarker candidates					
Colorectal cancer	Serum	LPC(16:0); LPC(18:2); LPC(18:1); LPC(18:0); LPC(20:4); LPC(22:6); PC(34:1); LPA(16:0); LPA(18:0); LPC(16:0)	Palmitic amide; Oleamide; Hexadecanedioate; Octadecanoate; Eicosatrienoate; Myristate	DI-FTICR-MS	[40]
**Research aim:** To discriminate colorectal cancer patients from controls by metabolomic biomarkers and to reveal the stage-related biomarkers for colorectal cancer and the changing trends of four lipid species in the colorectal cancer progression					
Hepatic cancer	Tissue	Arachidyl carnitine; Tetradecanal; Oleamide	β-Sitosterol; l-Phenylalanine; LPC(18:2); Glycerophosphocholine; LPE(18:3); Chenodeoxycholate glycine conjugate; LPC(22:6); Quinaldate; LPE(18:0); LPC(18:0); LPC(20:4)	LC-LTQ-Orbitrap-MS	[41]
**Research aim:** To select characteristic endogenous metabolites in hepatitis B virus-related hepatocellular carcinoma patients and to identify their molecular mechanism and potential clinical value					
Hepatic cancer	Urine	Octanedioate; Glycine; Tyrosine; Threonine; Butanedioate	Heptanedioate; Ethanedioate; Xylitol; Urea; Phosphate; Propanoate; Pyrimidine; Butanoate; Trihydroxypentanoate; Hypoxanthine; Arabinofuranose; Hydroxyproline dipeptide; Xylonate	GC-QMS	[42]
**Research aim:** To investigate the urinary metabolic difference between hepatocellular carcinoma patients and normal subjects and to find biomarkers for hepatocellular carcinoma					
Hepatic cancer	Serum	Cortisol; GCA; GCDCA; C16:1-CN; FAs (C16:1, C16:0, C18:2, C18:1, C18:0, C20:5, C20:4, C20:2, C22:6, C22:5)	Tryptophan; LPC(14:0); LPC(20:3); LPC(20:5); C10-CN; C10:1-CN; C8-CN; C6-CN	LC-Q-TOF/MS	[43]
**Research aim:** To study the related metabolic deregulations in hepatocellular carcinoma and chronic liver diseases and to discover the differential metabolites for distinguishing the different liver diseases					
Hepatic cancer	Plasma	LPC(24:0); Glycodeoxycholate; Deoxycholate 3-sulfate	LPC(14:0); LPC(16:0); LPC(18:0); LPC(18:1); LPC(18:2); LPC(18:3); LPC(20:4); FA(24:0); FA(24:1); LPC(20:2); LPC(20:3); LPC(20:5)	UPLC-QqQ/MSGC-QMS	[44]
**Research aim:** To evaluate the molecular changes in the plasma of hepatocellular carcinoma patients and to provide new insights into the pathobiology of the diseases					
Hepatic cancer	Feces	LPC(18:0); LPC(16:0)	Chenodeoxycholate dimeride; Urobilin; Urobilinogen; 7-Ketolithocholate	UPLC-Q-TOF/MS	[45]
**Research aim:** To find fecal metabolite biomarkers for distinguishing liver cirrhosis and hepatocellular carcinoma patients from healthy controls					
Pancreatic cancer	Saliva	Cadaverine; 2-Aminobutyrate; Alanine; Putrescine; Methylimidazole acetate; Trimethylamine; Piperidine; Leucine + Isoleucine; Phenylalanine; Tyrosine; Histidine; Proline; Lysine; Glycine; Ornithine; Burimamide; Ethanolamine; GABA; Aspartate; Valine; Tryptophan; β-Alanine; Glutamate; Threonine; Serine; Glutamine; Hypoxanthine; Choline; Carnitine	Taurine; Glycerophosphocholine	CE-TOF-MS	[21]
**Research aim:** To reveal the comprehensive salivary metabolic profiles of pancreatic cancer patients and healthy controls and to identify cancer-specific biomarkers with high discriminative ability					
Pancreatic cancer	Tissue	Taurine	Succinate; Malate; Uridine; Glutathione; UDP-*N*-Acetyl-d-glucosamine; NAD; UMP; AMP	UPLC-TOF/MS	[46]
**Research aim:** To investigate the differences in the metabolite profiles of normal and pancreas tumor tissue with a goal of developing prognostic biomarkers					
Pancreatic cancer	Serum	Lactate; Thiodiglycolate; 7-Hydroxyoctanoate; Asparagine; Aconitate; Homogentisate; *N*-Acetyl-tyrosine	Glycine; Urea, Octanoate; Glycerate; Decanoate; Laurate; Myristate; Palmitate; Urate; Margarate; Stearate	GC-QMS	[47]
**Research aim:** To evaluate the differences in the metabolomes between pancreatic cancer patients and healthy volunteers and to aid the discovery of novel biomarkers					
Pancreatic cancer	Serum	Arabinose; Ribulose	Valine; 2-Aminoethanol; *n*-Caprylate; Threonine; Nonanoate; Methionine; Creatinine; Asparagine; Glutamine; *o*-Phosphoethanolamine; Glycyl-Glycine; 1,5-Anhydro-d-glucitol; Lysine; Histidine; Tyrosine; Urate	GC-QMS	[48]
**Research aim:** To construct a diagnostic model for pancreatic cancer using serum metabolomics and to confirm its diagnostic performance					
Pancreatic cancer	Plasma	Arachidonate; Erythritol; Cholesterol; *N*-Methylalanine; Lysine; Deoxycholylglycine; Cholylglycine; LPC(16:0); Tauroursodeoxycholate; Taurocholate; LPC(18:2); PE(26:0); PC(34:2)	Glutamine; Hydrocinnamate; Phenylalanine; Tryptamine; Inosine	GC-TOF/MS LC-IT/MS LC-LTQ-Orbitrap-MS	[49]
**Research aim:** To seek novel metabolic biomarkers of pancreatic cancer					

In this review, we searched for the articles, in which the evaluations of differences between cancer and control were performed by metabolomics using gas chromatography-mass spectrometry (GC-MS), liquid chromatography-mass spectrometry (LC-MS), capillary electrophoresis-mass spectrometry (CE-MS), Fourier transform ion cyclotron resonance (FTICR-MS) and nuclear magnetic resonance (NMR), via PubMed database and so on, and their articles were shown in Table 1. In Table 1, the instruments used for metabolomics were described to specify the analytical method, and the aim of each article was also stated. In Table 1, upregulated or downregulated metabolites in serum/plasma, saliva, feces or urine of the cancer patients compared with healthy controls were listed. Regarding tissues, upregulated or downregulated metabolites in tumor tissues compared with non-tumor (normal) tissues in cancer patients were shown. Table 1 shows the list of metabolites that were demonstrated to be significantly changed between cancer and control in each article. In some articles, many metabolites with the significant alterations between cancer and control were exerted. Regarding these articles, only metabolites that were determined as biomarker candidates based on each criterion was listed in Table 1. Abbreviations: GABA, γ-Aminobutyrate; LPC, Lysophosphatidylcholine; PC, Phosphatidylcholine; LPA, Lysophosphatidate; LPE, Lysophosphatidylethanolamine; PE, Phosphatidylethanolamine; FA, Fatty acids; GCDCA, Glycochenodeoxycholate; GCA, Glycocholate; UDP, Uridine diphosphate; NAD, Nicotinamide adenine dinucleotide; UMP, Uridine monophosphate; AMP, Adenosine monophosphate; ATP, Adenosine triphosphate; CE-IT/MS, Capillary electrophoresis-ion-trap/mass spectrometry; CE-MS, Capillary electrophoresis-mass spectrometry; CE-TOF-MS, Capillary electrophoresis-time-of-flight mass spectrometry; DI-FTICR-MS, Direct infusion-Fourier transform ion cyclotron resonance-mass spectrometry; FTICR-MS, Fourier transform ion cyclotron resonance-mass spectrometry; GC×GC-TOF/MS, Two-dimensional gas chromatography-time-of-flight mass spectrometry; GC-QMS, Gas chromatography-quadrupole mass spectrometry; GC-TOF/MS, Gas chromatography-time-of-flight mass spectrometry; LC-IT/MS, Liquid chromatography-ion-trap/mass spectrometry; LC-LTQ-Orbitrap-MS, Liquid chromatography-linear ion trap quadrupole-Orbitrap-mass spectrometry; LC-QqQ/MS, Liquid chromatography-triple quadrupole/mass spectrometry; LC-Q-TOF/MS, Liquid chromatography-quadrupole-time-of-flight/mass spectrometry; NMR, nuclear magnetic resonance; QqQ-MS, Triple quadrupole-mass spectrometry; UPLC-QqQ/MS, Ultra performance liquid chromatography-triple quadrupole/mass spectrometry; UPLC-Q-TOF/MS, Ultra performance liquid chromatography-quadrupole-time-of-flight/mass spectrometry; UPLC-TOF/MS, Ultra performance liquid chromatography-time-of-flight/mass spectrometry.

### 3.4. Early Stage Cancer and Metabolomics

Early detection of cancer is very important for a complete recovery. Therefore, many researchers have searched for possible biomarkers of early cancer detection. In biomarker discovery research using metabolomics, evaluations of early cancer detection have been carried out. In the study by Kobayashi *et al.* a diagnostic model for pancreatic cancer was established using GC-MS-based serum metabolomics and multiple logistic regression analysis accompanied by the stepwise method [48]. This established model had a high sensitivity of 77.8% in resectable pancreatic cancer, namely relatively early stage pancreatic cancer, while sensitivities of CA19-9 and CEA were 55.6% and 44.4%, respectively. In serum lipid analysis for colorectal cancer, the metabolite profile data based on palmitic amide, oleamide, hexadecanedioate, octadecanoate acid, eicosatrienoate, LPC(18:2), LPC(20:4), LPC(22:6), myristate and LPC(16:0) exerted a sensitivity of 0.981 in early stage colorectal cancer patients compared to healthy volunteers [40]. In the analysis of plasma amino acids, alterations in levels of amino acids were observed in early stage lung, gastric, colorectal, breast, and prostate cancer [50]. Thus, the metabolites in biological fluids seem to be changed at the early stage of cancers, and metabolomics may be a powerful strategy for biomarker discovery, although detailed validation is still lacking at this point in time.

### 3.5. The Relationship between Metabolite Alterations and Cancer

Recently, studies aimed at biomarker candidate discovery based on amino acid-specific metabolite profiling have also been performed [50]. Moreover, it was demonstrated that high-mobility group box 1 protein (HMGB1) is released during the development and progression of colorectal cancer and subsequently induces muscle tissues to supply glutamine to cancer cells [51]. These findings suggest that increased HMGB1 levels lead to alterations in the blood amino acid profile and increased glutamine levels in colorectal tumors. In the paper by Miyagi *et al.* [50], the plasma level of tryptophan was significantly decreased in five types of cancer, i.e. lung, gastric, colorectal, breast and prostate cancer compared with healthy controls. Tryptophan is converted to kynurenine by indoleamine-2,3-dioxygenase, and it has been demonstrated that indoleamine-2,3-dioxygenase is over-expressed in cancer cells [52]. The possibility that indoleamine-2,3-dioxygenase may cause immune escape of various different tumors [52,53,54,55] has also been suggested, and that over-expression of indoleamine-2,3-dioxygenase in tumors may increase tryptophan metabolism, leading to a decreased of tryptophan in cancer patients. In addition, as shown in Table 1, the level of lactate seems to be upregulated in various gastroenterological cancers. Lactate is synthesized from pyruvate in the anaerobic condition, and it is known that this lactate synthesis is upregulated in cancer cells. This phenomenon is called “the Warburg effect” [56], so this reaction possibly promotes lactate synthesis, leading to an increased level of lactate. Thus, it seems that there are not only specific metabolite alterations in certain cancers but also common metabolite alterations in various cancers. These metabolite alterations are more likely to be reflected by the results of the metabolite biomarker candidates. Therefore, to draw firm conclusions about metabolite biomarker candidates for gastroenterological cancer it is important to understand the relationship between metabolite alterations and cancer, and moreover to elucidate the reasons for the observed alterations in the metabolite profile.

## 4. Future of Metabolomics-Based Disease Diagnosis

### 4.1. Procedures for Long-Term and Large-Scale Metabolomics Research

In the future, metabolomics is expected to be used in the clinical setting to screen for a variety of diseases including gastroenterological cancers. If metabolomics technology in screening programs enables early diagnosis, it can result in marked improvements in patients’ quality of life. Recently, the Human Serum Metabolome (HUSERMET) Consortium recommended the procedures for long-term and large-scale metabolomic studies involving thousands of human serum/plasma samples [57]. Subsequently, a method for the global metabolite profiling of animal and human tissues has also been proposed [58]. The HUSERMET Consortium recommended the methods for sample collection, sample preparation, and data acquisition for LC-MS- and GC-MS-based studies and also pointed out that the most important stage in large-scale metabolomic studies is appropriate sample collection, because the systematic failure at the beginning of the investigation could invalidate the whole study. In addition, they proposed the protocols for large-scale GC-MS-based metabolomic studies, which describe the number of samples that should be prepared each week and the number of samples that should be measured in a day and recommend the usage of the retention index instead of retention time. The use of standard operating procedures based on validated protocols is important for studies attempting to find novel metabolite biomarker candidates. Quality control and assurance (QC/QA) is also important in the long-term and large-scale metabolomic studies [57,59,60,61,62]. Samples for QC are analyzed every batch throughout all measurement batches, and signal intensity, peak shape, retention time, separation resolution, mass accuracy, and the amount of detectable peaks are checked by using data obtained from QC samples. The pooled biological fluid samples and standard compound mixture samples may be used as QC samples. Before starting the batch measurement, it may be required to confirm the status of the injector, the mass spectrometer, and so on. Instrument tuning including mass calibration and sensitivity check is also required routinely as well as after the instrument maintenance. Recently, the analyzing workflow for the large-scale non-targeted serum metabolite profiling by LC-MS was visualized in a PubMed-indexed video journal [63].

### 4.2. Sampling for Biomarker Discovery Research by Metabolomics

In the clinical setting, the analyses of biomarkers present in serum/plasma, saliva, urine, and tissue samples have been prevalent. However, collecting tissues is invasive, and therefore serum/plasma, saliva, and urine are well used. Especially serum/plasma biomarkers are preferable due to the ease of collection, and there are a variety of biomarker studies using serum/plasma. In the metabolomics research field, serum/plasma are also well used, and metabolomics-based studies evaluating the differences between plasma and serum metabolite levels have been carried out recently. In the report by Yu *et al.* [64], the serum levels of 104 of 122 metabolites were found to be about 10% higher than their plasma levels, and nine metabolites displayed serum levels that were more than 20% higher than their plasma levels. Yu *et al.* demonstrated that both plasma and serum metabolite data exhibited good reproducibility, but the plasma data displayed better reproducibility than the serum data. In another report by Yin *et al.* [65], it was stated that the exposure of blood to room temperature led to increased levels of hypoxanthine and sphingosine 1-phosphate, and hence, Yin *et al.* suggested that the following procedures should be employed during sample collection: the use of ethylenediaminetetraacetic acid (EDTA)-plasma samples is recommended for situations involving the metabolomic analysis of clinical samples; hemolyzed samples should be excluded; blood should be placed in ice water immediately after collection and should not be stored for longer than 2 h; the use of non-refrozen plasma is recommended (repeated freezing should not be performed), and MS data should be carefully examined for unexpected signals (the selection of blood collection tubes is also important because chemical noise derived from blood collection tubes can interfere with data analysis). In addition, the intra- and/or inter-day variance of metabolite levels has to be taken into consideration. Intra- and/or inter-day variance data has been reported for some metabolites [39,66,67]; for example, a previous study found that the tryptophan levels observed in the afternoon and at night were significantly lower than those detected in the morning. During pre-treatment and the subsequent measurement process, some metabolites might be unstable, and thus, it is necessary to confirm their corresponding metabolites and to eliminate any unstable metabolites from the subsequent analyses.

As for urine, the need to correct the obtained metabolite concentrations is an issue, although the correction using creatine and creatinine levels has been described in a great number of studies. However, collecting urine is non-invasive, and urine requires less sample pretreatment, because the protein level in urine is lower leading to a lack of complexity. Thus, urine has a number of advantages as an analytical material over other biological fluids [68,69,70], and in the future it may be recognized that urine is the most suitable biological fluids for the metabolomic approach to obtain meaningful diagnostic information.

Recently, metabolomic studies using saliva have also been carried out in the medical research field [20,21]. In humans, there are the three paired major salivary glands—the parotid gland, the submandibular gland, and the sublingual gland—and saliva is secreted from these major salivary glands. Saliva contains various DNAs, mRNAs, proteins including enzymes and antibodies, metabolites, and other molecules. Some of these molecules pass into the saliva from the blood stream via transcellular or paracellular routes. Therefore, saliva may correspond to blood regarding the reflection the physiological state of the body, and may be useful as a material containing disease biomarkers. Saliva collection is easy and noninvasive, and moreover, no specialized equipment is needed to obtain saliva [71]. Now, salivary diagnostics is recognized as one of the main approaches in biomedical basic and clinical areas [72], and it has been demonstrated that molecules in saliva may be associated with disease conditions [73,74,75]. To date, the number of metabolomics studies using saliva is small, but the potential of saliva metabolomics as a biomarker discovery approach has been proven by the accumulated results from saliva metabolomics.

### 4.3. Validation for Biomarker Discovery Research by Metabolomics

After discovering novel metabolite biomarker candidates whilst paying careful attention to the above issues, validation testing should be performed. In the disease biomarker discovery research, the use of samples obtained from other facilities is also important. The proposal by Yin *et al.* as shown above [65], lists considerable issues when the validation research is carried out in other facilities. In addition, during validation, it might be better to use different instruments from those utilized to detect novel metabolite biomarker candidates. Furthermore, techniques other than MS should be employed. When MS is used, it is necessary to prepare stable isotopes corresponding to the metabolite biomarker candidates if possible. The quantitative performance of mass spectrometers is affected by various factors such as ion suppression [76]. Therefore, stable isotopes are required to obtain detailed quantitative information about alterations in the levels of the target molecules [77]. Stable isotopes will also be essential for quantitative evaluations if the metabolomics-based research using MS is to result in practical clinical applications. During the validation process and in clinical practice, the use of multiple reaction monitoring (MRM) coupled with stable isotopes and triple quadrupole (QqQ) MS is a powerful method for measuring the levels of targeted metabolites, because MRM based on QqQ-MS leads to molecule detection with high sensitivity, selectivity, reproducibility as well as a broad dynamic analysis range. MRM coupled with stable isotope dilution using QqQ-MS is a longtime and principal method to quantify small molecules and also a powerful method for quantitative measurement of targeted proteins [78,79,80] Recently, an analysis of mouse blood metabolites using GC/QqQ-MS was validated, although the study did not use stable isotopes [81]. An metabolomic article describes the quantification of metabolites in serum/plasma carried out by LC-MS coupled with stable isotopes as internal standards, which are contained in the AbsoluteIDQTM p180 kit (BIOCRATES Life Sciences AG, Innsbruck, Austria) [82]. Through a strict validation process, the candidates can be narrowed down to several metabolites, and some biomarker candidates that exhibit high repeatability can be utilized for clinical application after assay optimization.

### 4.4. Assay Optimization of Mass Spectrometry-Based Metabolomics

Regarding the assay optimization of MS-based analysis systems, some problems remain to be resolved, for example, it would be useful if the following processes could be automated: (1) metabolite extraction; (2) the pre-treatment process; (3) data analysis including peak alignment, annotation, and identification; and (4) the output of the obtained results. Regarding the automation of metabolite extraction and the pre-treatment process, dried blood spot sampling, in which blood is blotted and dried on filter paper, and supercritical fluid extraction (SFE), which is an extraction technique involving the use of supercritical carbon dioxide, have been studied. When SFE is performed, it is not necessary to perform sample pre-treatment, and SFE is also suitable for extracting hydrophobic compounds. Recently, SFE was combined with MS, and the analysis of blood metabolites using this system accompanied by dried plasma spotting is currently being investigated [83]. Then, in biomarker research, the analysis of volatile organic compounds (VOCs), which include molecules such as alcohols, aldehydes, ketones, and other heterocyclic compounds, has been performed with combination of headspace-solid phase microextraction (HS-SPME) and GC-MS [84,85,86]. In this combination approach, the solvent extraction step for volatile analysis is not needed. Moreover, the analyzing system for VOCs in blood was constracted usng in-tube extraction (ITEX), which is superior to HS-SPME [87]. The systems using HS-SPME and ITEX do not need manual metabolite extraction from biological fluids, and so this may be useful for assay optimization. In addition, some studies have used an automated system for sample preparation before MS measurement [88,89]. Although the study regarding the automation of each process is ongoing, at present the metabolites are manually extracted via liquid-liquid and solid-phase extraction, and other processes are also largely performed manually.

### 4.5. Mass Spectrometry Data Preprocessing, Peak Alignment and Peak Identification

For automatic MS data preprocessing, peak alignment, and molecule identification, software for metabolome analysis are freely available or can be purchased. Examples of the software include MetAlign, XCMS, MZmine, AIoutput, and MRMPROBS [90,91,92,93,94]. For metabolite identification in metabolome analysis, the metabolite database including mass spectrum and retention time/retention time index may be used. Construction of an in-house database is needed, but METLIN and MassBank are also available for metabolite peak identification based on the fragment ion data [95]. To obtain the metabolite information such as the biological/biochemical characteristics and the related pathway, the Human Metabolome Database (HMDB), Kyoto Encyclopedia of Genes and Genomes (KEGG), Recon X and so on are convenient [96,97,98]. Especially, HMDB includes spectral data for human metabolites.

Thus, the technology for metabolome analysis is being innovated, the information is accumulated, resulting in development of the metabolomics-based biomarker discovery research. However, easier extraction, pretreatment, and data analysis methods are required to make metabolome analysis more practical. Therefore, it is hoped that an automated analysis system that performs all of the required processes from metabolite extraction to data output will be developed.

## 5. Conclusions

The Japanese population has the highest life expectancy in the world. Due to its aging society, the working population in Japan has been rapidly decreasing, and now the most populous age group is the 60-69-year-olds. As a result, the medical costs of elderly people in Japan have increased every year, and these increases are regarded as a financial problem for the national government. Therefore, the development of a low-cost and easy diagnostic approach for detecting diseases at an early stage is needed to reduce medical expenses. Similar problems have arisen in various developed countries. Recently, various types of clinical samples have been subjected to metabolome analysis using GC-MS, LC-MS, CE-MS, matrix-assisted laser desorption ionization (MALDI)-MS, NMR spectrometry, or FT-IR spectrometry in order to discover novel biomarkers and elucidate the onset mechanisms of diseases. It is important to obtain disease-specific metabolome profiles in order to increase our understandings of diseases. Novel findings based on these disease-specific metabolome profiles are useful not only for discovering new biomarkers and elucidating the onset mechanisms of diseases, but also for developing novel therapeutic strategies, although accomplishing these aims will probably require the integration of omics data obtained from genomics-, transcriptomics-, and proteomics-based approaches as well as data acquired using metabolomics. The metabolomics-based research will hopefully increase our understanding of various diseases and lead to the elucidation of novel metabolite biomarkers. In addition, the development of metabolomics-based screening processes that only require a single drop of blood and allow diseases to be diagnosed at an early stage is greatly desired.
